# What is the level of safety culture in French nursing homes? The EHPAGE study

**DOI:** 10.1186/s12913-021-07336-w

**Published:** 2021-12-11

**Authors:** Delphine Teigné, Guillaume Mabileau, Leila Moret, Noémie Terrien

**Affiliations:** 1 QualiREL Santé, Hôpital Saint Jacques, 85 rue Saint Jacques, 44093 Nantes, France; 2 Public Health Department, University Hospital of Nantes, 85 rue Saint-Jacques, 44093 Nantes Cedex 1, France; 3 UMR INSERM U1246-SPHERE “methodS for Patients-centered outcomes & HEalth REsearch”, University of Nantes, University of Tours, 22 Boulevard Benoni Goullin, 44000 Nantes, France

**Keywords:** Nursing homes, Safety culture, Risk management, Measurement, Quality improvement, Survey, Factor

## Abstract

**Background:**

French nursing homes (NHs) are in the early stages of implementing their risk management approach. The latter includes the development of a safety culture (SC) among professionals. A training package to support NHs in implementing a risk management strategy has been designed by QualiREL Santé, a regional body that provides support in quality and risk management. The aim is to improve SC. No data are available about the level of SC in French NHs. This study evaluates the level of SC and identifies predictors of SC scores in NHs that will subsequently benefit from the training package.

**Method:**

The study was proposed to NHs who are members of QualiREL Santé in 2 French departments. Inclusion criteria were voluntary participation, the commitment of top management to benefit from the training package, and the absence of previous risk management support provided by QualiREL Santé. The NHSOPS-F questionnaire (22 items measuring 7 dimensions of SC) was administered to professionals between January and March 2016. 14 variables related to the structural profile of the NHs and the strategic choices of top management in terms of healthcare safety were recorded. Scores for 7 dimensions were calculated for all of the included NHs. Further modelling identified predictive factors.

**Results:**

58 NHs were included. The response rate for the NHSOPS-F (*n* = 1946 professionals) was 64% (Q1-Q3 = [49.4;79.0]). Staffing was the least-developed dimension (11.8%), while scores were highest for Feedback and communication about incidents (84.8%). Being attached to a public hospital was associated with poorer perceptions of SC, notably for the dimension “Overall perceptions of resident safety and organizational learning” (β = − 19.59;*p*-value< 0.001). A less-developed SC was also significantly linked to existing Quality initiatives.

**Conclusions:**

Overall, French NHs must prioritise issues of staffing, teamwork and compliance with procedures. The role of human factors within teams should be exploited by top management. Our initial findings will help to adapt improvement approaches and are particularly relevant to local and national policies during the ongoing pandemic.

**Supplementary Information:**

The online version contains supplementary material available at 10.1186/s12913-021-07336-w.

## Introduction

In France, as in other countries, safety has been a public health concern for many decades in health care facilities. More recently, attention has turned to nursing homes (NHs). Initiatives to improve care quality and safety in facilities generally include the promotion of a patient safety culture (SC) among professionals [1].

SC is a multidimensional concept, and there is currently no consensus on the definition, number, nature and denomination of its dimensions [2]. The European Society for Quality in Health Care defines it as, “an integrated pattern of individual and organisational behaviour, based upon shared beliefs and values that continuously seeks to minimise patient harm, which may result from the processes of care delivery” [3]. According to Shortell et al., an assessment of healthcare teams’ SC is essential for needed to reach objectives in terms of quality and risk management (RM) in healthcare delivery systems [4]. This assessment helps to raise staff awareness of issues related to patient safety, and to identify potential areas for improvement.

The Hospital Survey on Patient Safety Culture (HSOPSC) and the Nursing Home Survey on Patient Safety Culture (NHSOPS) questionnaires were developed by the Agency for Healthcare Research and Quality (AHRQ) [5, 6]. Each measures 12 dimensions of SC and they have been translated and transculturally adapted many times throughout the world [7–11]. In France, the validated psychometric adaptation of the HSOPSC resulted in an instrument with 10 dimensions [12]; and the French version of the NHSOPS (called NHSOPS-F) measures 7 dimensions of SC [13]. Questionnaires can be used to assess the impact of initiatives designed by research teams to improve SC [14, 15]. Initiatives include interventions such as team training, the creation of patient safety teams, or patient safety education programmes [16], and may target one or more dimensions of SC. They are strategic choices with respect to healthcare safety. Recently, Xie et al. measured the impact of a 76-h RM and SC training programme for nurses in Chinese hospitals [14]. In other assessments, the collection of additional elements has made it possible to study parameters that influence certain dimensions of SC scores [14, 17, 18]. Banaszak et al. [17] used data from American NHs cohorts to study the relationship between the legal status of NHs and SC scores. Cappelen et al., investigated the link between SC and organisational initiatives launched by Norwegian top management [19]. Finally, in Norway, Titlestad et al. [18] examined perceptions of SC in NHs, and links with respondents’ level of education, their knowledge of diabetes management guidelines, and medical data for their own diabetic residents.

In France, the National Patient Safety Programme 2013–2017 addressed the challenges of developing a SC based on a RM approach [20]. In 2016, the government created several *Structures Régionales d’Appui*, whose mission is to contribute to improving the quality of healthcare and patient safety [21]. Their role is to work with professionals in hospitals and medico-social structures, including NHs [21]. At the same time, the literature highlights that there is a lack of data about SC in French NHs and there is a long way to go before they implement the RM approach [22]. In this context, QualiREL Santé (Regional Support Structure for Quality and Safety of Care) designed a training package to support NH in the implementation of their RM system. It is therefore important to understand the levels of SC in French NHs and the impact of the implementation of the training package. The study is part of the EHPAGE research project. The project was financed by the General Directorate for Healthcare Provision (direction générale de l’offre de soins) (2015–2017). The trial registration number is NCT02908373 (September 21, 2016; “Retrospectively registered”).

The main objective of this article is to describe the perceptions of SC, and the associated factors, of professionals working in the selected NHs. The aim was therefore to: (i) evaluate the level of SC; and (ii) to identify factors that could predict SC scores. The results provide new insight into what can be done to improve SC in NH.

## Method

### Target institutions

In France, the accommodation provided to dependent elderly people who require day-to-day care has undergone many changes. French NHs accommodate elderly people who have lost their physical and/ or mental independence, and who can no longer live in their own home [23]. Medical and paramedical care is provided by staff employed directly by the NH (care assistants, nurses, etc.) and external professionals who are contracted by the facility (local doctors, physiotherapists, chiropodists, etc.). The establishment offers assistance in daily activities (cares, help in getting out of and going to bed, getting washed, meals, etc.) and services such as catering, laundry and entertainment. The legal status of French NH can vary (attached to a public hospital, independent, or part of a group) [24]. They also differ in terms of number of beds and the medical facilities they offer. High-dependency residents are scored according to their degree of dependency, and are accommodated in a specialised unit [24]. Another difference is the number of staff, and the ratio of staff to residents.

The first inclusion criterion for NHs was being a member of QualiREL Santé. NHs were so located in 2 french *départements*: Loire-Atlantique and Vendée (a *département* is an administrative division of France). Other inclusion criteria were their willingness to use the QualiREL Santé support tool to implement a RM system, a commitment from top managers to implement the RM approach, and that no support had been provided by QualiREL Santé on the topic of RM upstream of the project. Inclusion was conducted in September 2015.

### Data collection

#### Collection of descriptive variables of NHs

14 descriptive variables des NHs participants were collected at the beginning of the project. 7 variables related to the structure of NH: geographical location, bed capacity, legal status, being part of a group or not, the existence of specialised units, the average Dependency score, and the staff/ resident ratio. 7 other variables documented top managers’ strategic choices in terms of safety of care: existence of an established policy of ongoing improvement in Quality and RM, the existence of other strategic plans put in place by the establishment, and the development of a Quality and RM policy, facilitated by the presence of a designated RM Quality officer, a qualified RM graduate, or an external Quality and RM service provider. The categorizations of the variables are presented in Table 1. The tools allowing the collection of descriptive variables were developed specifically by the research team. They were without license.

**Table 1:** Descriptive statistics for nursing homes

Descriptive variables in 2016 (*n* = 58)	n (%) or mean (SD)
Geographic location *	
Loire Atlantique	31 (53.4%)
Vendée	27 (46.6%)
Beds*	
≥80	28 (48.3%)
< 80	30 (51.7%)
Legal status *	
Private or public, independent or regional	44 (75.8%)
Attached to a public hospital	14 (24.2%)
Specialised units *	
No	28 (48.3%)
Yes	29 (50%)
NA	1 (1.7%)
Part of a group *	
No	26 (44.8%)
Yes	21 (36.2%)
Hospital-based	11 (19.0%)
Dependency score*	
Mean (SD)	621.9 (82.77)
NA	2 (3.4%)
Staff/ resident ratio *	
*Mean (SD)*	0.80 (0.37)
*NA*	0 (0.0%)
RM officer **	
No	22 (38%)
Yes	35 (60.3%)
NA	1 (1.7%)
Qualified RM expert **	
No	32 (55.2%)
Yes	25 (43.1%)
NA	1 (1.7%)
External Quality and RM officer **	
No	56 (96.6%)
Yes	1 (1.7%)
NA	1 (1.7%)
Established policy of ongoing improvement in Quality and RM **	
No	22 (53.8%)
Yes	35 (60.3%)
NA	1 (1.7%)
Other strategic plans put in place by the NH**	
No	31 (53.5%)
Yes	26 (44.8%)
NA	1 (1.7%)
Active Quality improvement approach **	
No	6 (10.3%)
Yes	51 (88%)
NA	1 (1.7%)
Active RM policy **	
No	21 (36.2%)
Yes	36 (62.1%)
NA	1 (1.7%)

M: Risk Management NA: no answer SD: Standard Deviation

* Variables related to the structural profile of NH ** Variables related to strategic safety of care choices

##### Measuring staff perceptions of SC: variables and the administration of the NHSOPS-F questionnaire

The French version of the psychometrically-validated NHSOPS [13] includes 22 items that measure the following seven dimensions of SC: Overall perceptions of resident safety and Organisational learning; Handoffs; Teamwork; Supervisor expectations and actions promoting resident safety; Compliance with procedures; Staffing; and Feedback and communication about incidents (Table 2). The content is presented under four headings: “work in your facility”, “communication”, “your hierarchy” and “your facility”. The self-administered questionnaire is aimed at professionals working in French NHs. The 22 items are scored on a 5-point Likert scale (never, rarely, sometimes, most of the time, always – or – do not agree at all, do not agree, agree up to a point, agree, completely agree). “Does not apply” and “Don’t know” are further response categories. Finally, it includes items relating to the socio-demographic characteristics of respondents. The tool does not require license for its use.

**Table 2:** Respondent characteristics for the survey (*n* = 1946)

	French survey	
	n	%
Professional fields		
Paramedical	822	42.2
Administration/logistics/technical	166	8.6
Educational/ Psycho-social	49	2.5
Doctor	55	2.8
Others	41	2.1
Do not wish to answer/missing data	813	41.8
Age bracket		
Under 25 years old	106	5.5
26 to 35 yrs. old	382	19.6
36 to 45 yrs. old	467	24.0
46 to 55 yrs. old	480	24.7
Over 56 yrs. old	139	7.1
Do no wish to answer/missing data	372	19.1
Number of years working in a nursing home		
Less than11 months	163	8.4
1 to 5 years	465	23.9
6 to 10 years	344	17.7
11 years or more	495	25.4
Do not wish to answer/missing data	479	24.6
Weekly working hours in a nursing home		
15 h or fewer	122	6.3
16 to 24 h	128	6.6
25 to 35 h	879	45.1
36 h or more	290	14.9
Do not wish to answer/missing data	527	27.1

The NHSOPS-F questionnaire was administered from January to March 2016. All salaried professionals working in NHs (not only those directly involved in resident care) were invited to participate by an appointed contact point in each facility. This contact point was a professional chosen by the NH’s management, and this person was in charge of the deployment of the process. Private sector professionals who were working in the NH were also included if they spent at least 10% of their time there (i.e. half a day per week). In fact, the AHRQ recommends including private sector professionals in the survey even if they only spend a few hours a week in the NH [6]. The threshold of 10% was chosen as it is sufficient for professionals to be integrated into the structure of the NH, interact with permanent salaried professionals, and be sufficiently acquainted with the NH to respond to the survey.

The survey was conducted over a six-week period, divided into the four steps recommended in the AHRQ user’s guide [6]: 1) publicity and promotion; 2) distribution; 3) issue of reminders; and 4) data collection. All professionals received a copy of the questionnaire accompanied by a cover letter in which the purpose of the study was explained. They were asked to return the questionnaire anonymously to a drop-box in the facility [13]. All of the questionnaires were centralized by QualiREL Santé in order to record them and check data anonymity. Responses were recorded by an external technician, and data capture was blind, in duplicate with cross-checking [13].

### Data analysis

Descriptive statistics were performed for all variables. Qualitative variables were expressed as percentages, and quantitative variables were expressed as mean with standard deviation.

As in the original American version [6], subscale scores were calculated as the mean percentage of positive responses on all items in each dimension (excluding missing responses and “does not apply” or “don’t know”). The responses “completely agree/agree” and “most of the time/always” for positively-worded items, and “do not agree at all/do not agree” and “never/rarely” for negatively-worded items were classified as positive responses. A dimension was said to be “underdeveloped” if the score was < 50%; “developed” if it was > 75% and “developing” if it was between 50 and 75% [6, 13].

We analyzed each of the seven SC scores in 2016. Linear regression models were used to assess variables associated with each SC scores in univariate analyses to identify potential predicting factors with a significance level alpha of 0.2. Multivariate analyses were then performed according to the backward stepwise method in order to identify models that best fit the data, and keep the most relevant factors associated to each SC scores and their evolution with a significance level alpha of 0.05. Validity and goodness of fit of the models was checked using the adjusted R^2^ and the F-test. All statistical analyses were performed using R software (version 4.0.0).

### Ethics approval and consent to participate

The EHPAGE research protocol was approved by the Advisory Committee on Health Research Information Processing, and an ethics committee (“Groupe Nantais d’Ethique dans le Domaine de la Santé “GNEDS - University Hospital of Nantes). The participation of professionals in the research was the subject of prior information. Participation was voluntary and informed consent was implied upon completion of the questionnaire (procedure approved by the ethics committee). According to articles L1121–1 and R1121–2 of the French Public Health Code, Institutional Review Board (IRB) approval was not necessary.

## Results

### Nursing home included characteristics

The NHs included were 58. Table 1 documents the 14 descriptive variables for these NHs. Fourteen were attached to a public hospital, while the remainder were either independent or regional, private or public facilities. Capacity was below 80 beds in 30 cases. RM approaches were either the responsibility of a qualified professional (an employee or external contractor) or a RM officer (26 and 35 NHs respectively). A Quality improvement approach was reported to have been initiated in 51 NHs; RM procedures were in place at 36.

### Participation of professionals

The administration of the questionnaire targeted 3390 professionals. The number of professionals who responded was 1946. The median participation rate by NH was 64% (Q1-Q3 = [49.4;79.0]). 69.1% of the respondents were working in contact with the residents. Almost half of the respondents (42.2%) belonged to the paramedical field (Table 2). The age of the professionals was > 35 for 55.8%. Half the respondents had worked in the profession for under 10 years (50.0%). Weekly working hours were 25 to 35 h for the majority (Table 2).

### Professionals’ perception of resident safety culture

Table 3 shows scores for the seven SC dimensions and 22 items, for the 58 NHs. At the beginning of the project, one dimension (Staffing) was underdeveloped, two dimensions (Teamwork and Compliance with procedures) were developing, and the other four dimensions were developed.

**Table 3:** SC scores by dimension and item (n = 58 NHs)

Dimensions / Items	SC score (%)	
	mean	[Q1; Q3]
***Dimension 1***: ***Overall perceptions of resident safety / Organizational learning***	***75.8%***	**[69.5%; 87.8%]**
D6 – This nursing home does a good job keeping resident safe	81.6%	[76.8%; 91.9%]
D8 – This nursing home is a safe place for residents	88.6%	[82.4%; 97.1%]
D4 – It is easy to make changes to improve resident safety in this nursing home	62.7%	[50.0%; 77.1%]
D5 – This nursing home is always doing things to improve resident safety	77.5%	[71.2%; 91.2%]
D10 – When this nursing home makes changes to improve resident safety, it checks to see if the changes worked	68.4%	[55.9%; 82.3%]
***Dimension 2: Handoffs (transfer of information)***	**76.9%**	**[72.0%; 84.2%]**
B1 – Staff are told what they need to know before taking care of a resident for the first time	77.7%	[72.5%; 86.9%]
B2 – Staff are told right away when there is a change in a resident’s care plan	80.6%	[71.8%; 89.7%]
B3 – We have all the information we need when residents are transferred from the hospitals	64.9%	[58.3%; 72.3%]
B10 – Staff are given all the information they need to care for residents	84.6%	[80.9%; 91.0%]
***Dimension 3: Teamwork***	**60.4%**	**[48.8%; 72.4%**]
A1 – Staff in the nursing home treat each other with respect	61.2%	[48.9%; 74.3%]
A2 – Staff support one another in this nursing home	52.8%	[39.2%; 68.1%]
A5 – Staff feel like they are part of a team	70.8%	[60.0%; 82.1%]
A9 – When someone gets really busy in this nursing home, other staff help out	56.7%	[46.0%; 62.5%]
***Dimension 4: Supervisor expectations and actions promoting resident safety***	**79.2%**	**[72.7%; 87.4%]**
C1 – My supervisor listens to staff ideas and suggestions about resident safety	82.6%	[76.1%; 91.4%]
C2 – My supervisor says a good word to staff who follow the right procedures	75.9%	[69.2%; 85.7%]
***Dimension 5: Compliance with procedures***	**55.0%**	**[46.0%; 62.9%]**
A6R – Staff use shortcuts to get their work done faster	45.9%	[36.7%; 56.0%]
A14R – To make work easier, staff often ignore procedures	64.1%	[52.5%; 71.7%]
***Dimension 6: Staffing***	**11.8%**	**[3.7%; 17.7%]**
A3 –We have enough staff to handle the workload	13.9%	[3.1%; 20.6%]
A8R – Staff have to hurry because they have too much work to do	9.6%	[2.0%; 14.2%]
***Dimension 7: Feedback and communication about incidents***	**84.8%**	**[79.3%; 91.9%]**
B5 – In this nursing home, we talk about ways to keep incidents from happening again	80.5%	[72.5%; 89.5%]
B6 – Staff tell someone if they see something that might harm a resident	92.4%	[90.1%; 96.3%]
B8 – In this nursing home, we discuss ways to keep residents safe from harm	81.4%	[75.0%; 93.0%]

SC: Safety Culture NH: nursing home [Q1;Q3]: first and third quartiles

SC < 50%: underdeveloped dimension 50% ≤ SC < 75%: developing dimension SC ≥ 75: developed dimension

NOTE: items flagged R: response scores are reversed to preserve the negative meaning

### Relationship between descriptive variables of nursing home and perception of safety culture

The results of the final multivariate models are presented in Table 4. Details of the univariate models used in their construction are available in Table A1 (Supplementary Material).

**Table 4:** Parameters included in the multivariate models that explain scores for the safety culture dimensions (n = 58 NHs)

Parameter		ßCoef. [95%CI]
*Dimension 1: Overall perceptions of resident safety and Organizational learning*		*adjusted R*^*2*^ *= 29.2%*
Public hospital	No	ref.
	Yes	− 19.59 [− 27.35;-11.82]**
*Dimension 2: Handoffs*		*adjusted R*^*2*^ *= 14.7%*
Public hospital	No	ref.
	Yes	− 8.66 [− 14.6;-2.72]*
Established policy of ongoing improvement in Quality and RM careful this separation is not homogeneous	No	ref.
	Yes	−4.73 [−9.98;0.52]
*Dimension 3: Teamwork*		*adjusted R*^*2*^ *= 24.2%*
Active RM policy	No	ref.
	Yes	−10.49 [− 18.73;-2.25]*
External Quality and RM officer	No	ref.
	Yes	−31.08 [−61.21;-0.94]*
Geographic location	Loire-Atlantique	ref.
	Vendée	11.35 [3.32;19.37]*
*Dimension 4: Supervisor expectations and actions promoting resident safety*		
N/A		
*Dimension 5: Compliance with procedures*		*adjusted R*^*2*^ *= 23.7%*
Beds	< 80	ref.
	≥ 80	−8.48 [−14.65;-2.32]*
Qualified Quality and RM officer	No	ref.
	Yes	−11.43 [−17.63;-5.24]**
External Quality and RM officer	No	ref.
	Yes	−27.91 [−51.31;-4.51]*
*Dimension 6: Staffing*		*adjusted R*^*2*^ *= 7.5%*
Active Quality improvement approach	No	ref.
	Yes	−10,85 [−19,88;-1,82]*
*Dimension 7: Feedback and communication about incidents*		*adjusted R*^*2*^ *= 14.9%*
Membership of a group	No	ref.
	Yes	−1.95 [−7;3.11]
	Attached to a public hospital	−8.58 [−14.83;-2.33]*
Active Quality improvement approach	No	ref.
	Yes	−7.48 [−14.92;-0.04]

*Significant at 5% **Significant at < 0.1%

NA: not analysable RM: Risk Management CI: Confidence Interval

Scores for Dimension 1 (Overall perceptions of resident safety and organisational learning) and Dimension 2 (Handoffs) were significantly lower in NH attached to public hospitals than in those with another legal status (β-coefficient = − 19.59/− 8.66; 95%CI = [− 27.35;− 11.82]/[− 14.60;− 2.72], respectively). Scores for Dimension 3 (Teamwork) were lower among NH in Loire-Atlantique; these establishments had already initiated a RM approach, and were using an external Quality and RM contractor (β-coefficient = − 11.35/− 10.49/− 31.08; 95%CI = [− 19.37;− 3.32]/[− 18.73;− 2.25]/[− 61.21;− 0.94]). Scores for Dimension 5 (Compliance with procedures) were lower among NH with over 80 beds, those with a qualified, in-house risk manager, and those that used an external Quality and RM provider (β-coefficient = − 8.48/− 11.43/− 27.91; 95%CI = [− 14.65;− 2.32]/[− 17.63;− 5.24]/[− 51.31;− 4.51]). Scores for Dimension 6 (Staffing) were lower among NH that had initiated a Quality improvement approach (β-coefficient = − 10.85; 95%CI = [− 19.88;− 1.82]). Scores for Dimension 7 (Feedback and communication about incidents) were lower for NH attached to a hospital than for those that were part of a group (β-coefficient = − 8.58; 95%CI = [− 14.83;− 2.33]).

## Discussion

### Review of SC scores

Evaluating the current SC contributes to raising the awareness of NH staff to issues related to residents’ safety, and gives them the opportunity to identify their strengths and weaknesses. In this study, 4 dimensions proved to be well-developed: “*Overall perceptions of resident safety and organizational learning*” (with a positive response rate of 75.8%), “*Handoffs*” (76.9%), “*Supervisor expectations and actions promoting resident safety*” (79.2%), “*Feedback and communication about incidents*” (84.8%). The majority of the professionals surveyed supported the idea that overall, the level of care provided was high quality, and that residents were safe. Globally, the information that was provided (whether he or she was new to the job, or whether there was a change in the care plan) was considered to be comprehensive and able to support a satisfactory level of care. Professionals also reported that they received frequent praise from top management, and that their suggestions for improving the safety of care were taken into account. They perceived that incidents were routinely addressed in their establishment, leading to a concerted search for solutions and an evaluation of any changes to be made. The dimensions “*Teamwork*” (60.4%) and “*Compliance with procedures”* (55.0%) were both developing. *“Staffing”* (11.8%) was the domain with the greatest potential for improvement, and the results underlined the lack of staff and the heavy workload.

Like our results, scores for the dimension *“Feedback and communication about incidents”* are highest in several other studies that have used the NHSOPS (83% in an American evaluation [15]; from 85.7 to 87.2% for 3 European evaluations [7, 19, 25]). “Staffing” was also the least developed (52% in the American evaluation [15]; 37.6 and 45% for, respectively, Belgian [25] and Norwegian [19] evaluations). The Swiss psychometric study of the questionnaire highlighted the dimension “Workload” (rather than “Staffing”). This dimension also had the lowest score (20% [7]). A qualitative evaluation of SC in ten of the 58 French NHs was conducted at the beginning of the EHPAGE study (the results have not been published). This highlighted a strong oral culture among professionals involved in providing personal care and support for residents. We see here a parallel between communication (in the broad sense of the word as used by professionals) and results for the dimension “*Feedback and communication about incidents”*. We must not forget that the implementation of the formal RM system will require a change in practices, with a move to written reports of incidents. Concerning the “*Staffing”* dimension, the literature confirms that this theme is one of the most common challenges in hospitals and NHs [14].

In France, results for dimensions that are found to be developed are similar to those of other studies; this is illustrated by, for example “*Supervisor expectations and actions promoting resident safety*” (79.2% in our study versus 75 to 87% in other studies [7, 15, 19, 25]. The dimension “*Compliance with procedures*” was also found to be less-well-developed in these same studies. The French score for the dimension *“Teamwork* “(60.4%) was lower than the score for the 3 European studies (from 76 to 80% [7, 19, 25]) but was close to the score in the American study (68% [15]). There is a clear link between perceptions of the difficulty of complying with procedures when the workload is high and staffing levels are low. It is possible that the dimension Teamwork could be overestimated by healthcare professionals, as highlighted in a recent report by the *Haute Autorité de Santé* [26]; this perception is all the more relevant as Teamwork is often a contributing factor in adverse events (AE) that occur [26]. It should also be borne in mind that several aspects make the comparison of international results difficult: notably, heterogeneity between the characteristics of NHs in different countries; differences in the adaptation of the questionnaires, and the design of the intervention [27].

### Predictors of SC scores

Predictive factors of SC scores (structural with respect to NHs and strategic for top management) were studied.

Having the status of public hospital or being attached to a hospital was associated with poorer perceptions of SC, notably for the dimensions “*Handoffs*”, “*Overall perceptions of resident safety and organizational learning”*, “*Feedback and communication*”. Bed capacity of over 80 was linked to a poorer perception of SC for the dimension “*Compliance with procedures*”. It is possible that the high number of actors in large facilities and the size of certain NHs leads to poor communication between departments and professionals (or at least a perception of it).

A less-developed SC was also linked to existing quality and/ or RM initiatives, and the presence of an external service provider or an in-house, qualified RM specialist. We can hypothesise that professionals who took part in the survey had a critical understanding of the situation in their NH, they understood the vocabulary that was used because they had been made aware of RM issues (by an external service provider or an in-house, qualified RM professional). A second hypothesis relates to the delegation of responsibility for quality and RM in French hospitals and NHs. It is possible that top management have assigned responsibility for these topics to professionals in order to comply with regulations, while at the same time failing to acknowledge their role in supporting change. The distinction between prescribed work and real work would explain our results regarding teamwork, staffing, compliance with procedures and communication about incidents. Finally, in some NHs, responsibility for RM has been given to an appointed member of staff. No relationship was found between this variable and SC scores. If there had been one, we would have been able to examine the qualifications of this person with regard to the theme (for example, a nurse or a member of the administration) or the time allocated by the NH to the function.

Some authors report the link between SC and the characteristics of professionals (their function, seniority, training) [28]. The high percentage of paramedical professionals in French NHs (42.2% of respondents in our study) and the general reluctance to provide socio-demographic data, prevented this analysis. The link between the structural profile of NHs and SC has been reported by Banaszak et al. [17], who found that legal status was a predictor of SC scores. Non-profit NHs had the lowest SC scores [17]. The inclusion of more independent and regional NHs versus private NHs would have allowed us to explore this specific legal status as a predictor. Finally, Cappelen et al. considered the link between SC and organizational initiatives set out by top management [19]. These initiatives consisted of the implementation of measures to improve patient safety, including an AE reporting system and staff training. The results underline that organisational initiatives, tailored to local needs, are predictors of SC. Top management can therefore create and support an environment in which staff feel responsible for resident safety by improving communication and participating in decision-making [19]. The authors caution, however, that significant effort is required to achieve improvement in SC.

### Strengths, limitations and perspectives

The results from the present study are the first concerning SC in French nursing homes. This evaluation provides a valuable insight into how SC is perceived by professionals who provide personal care to residents. Their feedback is important, because the literature underlines that managers’ perceptions of SC are often very positive [29]. These initial results show an overall moderate level of SC. As with any initial measurement in a NH, the results must be interpreted with care. Not all professionals are familiar with the vocabulary of RM or its approaches, which are recent in France [20]. Although the response rate to the questionnaires (64%) is close to that of other studies (66% [7], 69% [30]), it may constitute a selection bias. Because of the mode of administration of the questionnaires, reasons for non-participation in our study are not known. A study of the predictors of participation in evaluation campaigns would be useful in this context, and the results should be taken into account in future research [15]. The voluntary participation of NHs, and the fact that only two French *départements* were represented, means that the results cannot be extrapolated to the entire country. A national French survey will be conducted at the end of 2021 to further explore the local application of national policies. Moreover, SC predictors merit further, in-depth investigation, in order to extend the interpretation of our results and to tailor improvements. For example, the role of human factors in the team is not sufficiently explored by NHs, and several studies indicate that there is a need to investigate the relationship between teamwork, error reporting and learning from AEs in more depth [31, 32]. In teams where professionals trust each other and feel able to talk about their mistakes, AE reporting can be contradictory, either developed or, on the contrary, poorly developed. For one team, AE reporting is the logical outcome of their discussions [31]; for another, it is no longer relevant as the error has already been discussed [32]. In such a context, the implementation of the QualiREL Santé training package, which is specifically aimed at developing an AE reporting system, is particularly relevant. Finally, a key challenge is to study the (transformational) leadership of top management [19, 33]. The latter is cited as an essential factor for the development of SC. Seljemo et al. discuss the positive effect of transformational leadership on the job satisfaction of professionals, which, in turn, seems to reduce the frequency of AE and improve the quality of care [33].

## Conclusion

Faced with the huge challenge of an ageing population, the care pathway and inevitable changes to the care of NH residents, it seems unavoidable that political decisions must adopt a systemic approach to care safety [34]. This study highlights the strengths and weaknesses of French NHs with respect to the safety of residents. Overall, French NH must prioritise issues of staffing, teamwork and compliance with procedures. The legal status of the NH, or its membership of a parent group, along with its current organisation in terms of quality and/or RM are characteristics that are closely linked to SC scores. This study also underlines the challenge of developing an integrated approach that addresses both the quality and safety of care, and the quality of life at work. Managers (both specialists and generalists) must be convinced of the need for a SC and lead in a way that can sustain the culture in the long term. A better understanding of its specific needs has enabled each participating facility to develop a tailored improvement strategy and to launch targeted interventions. A training package has been developed to support NHs in implementing their RM approach. This tool, which is all the more interesting in the context of the current pandemic, should, ultimately, improve resident care by developing a SC among professionals. It is clearly time to develop a better understanding of the impact of this training package and the conditions for its successful implementation. The next set of results from the EHPAGE study are currently in press.

## Supplementary Information

**Additional file 1.**

## Data Availability

The authors agree to share the data. Please contact the corresponding author should you require any of the results from this study.

## Abbreviations

AE: Adverse events
AHRQ: Agency for Healthcare Research and Quality
HSOSC: Hospital Survey on Patient Safety Culture
NH: Nursing Home
NHSOPS: Nursing Home Survey on Patient Safety Culture
RM: Risk Management
SC: Safety Culture
