# Supplementary material for: What is the level of safety culture in French nursing homes? The EHPAGE study
Source: BMC Health Serv Res. 2021 Dec 11;21:1332. doi: 10.1186/s12913-021-07336-w (PMC8666034; doi:10.1186/s12913-021-07336-w)
Supplement: Supplementary file 1 — Additional file 1. [file 12913_2021_7336_MOESM1_ESM.pdf]

Table A1: Details of univariate and multivariate models explaining safety culture scores for the 7 dimensions in 2016 as a function of parameters (n=58 nursing homes)

		**Dimension 1**		**Dimension 2**		**Dimension 3**		**Dimension 4**	
		Univariate	*Multivariate*	*Univariate*	*Multivariate*	*Univariate*	*Multivariate*	*Univariate*	*Multivariate*
Geographic location	Loire-Atlantique	REF.		REF.		REF.		REF.	
	Vendée	1,91 [-6,06;9,87]		3,68 [-1,79;9,15]		11,41 [3,05;19,76]*	**11,35 [3,32;19,37]***	1,57 [-5,66;8,8]	
Beds	< 80	REF.		REF.		REF.		REF.	
	≥ 80	-1,38 [-9,34;6,58]		-2,63 [-8,13;2,87]		-9,71 [-18,2;-1,23]*		-5,97 [-13,02;1,09]	
Legal status	Private ou public, independant or regional	REF.		REF.		REF.		REF.	
	Attached to a public hospital	**-19,59 [-27,35;-11,82]****	**-19,59 [-27,35;-11,82]****	-9,06 [-15,08;-3,03]*	**-8,66 [-14,6;-2,72]***	-7,42 [-17,58;2,74]		-3,67 [-12,06;4,71]	
Specialised units	No	REF.		REF.		REF.		REF.	
	Yes	-0,68 [-8,72;7,37]		1,09 [-4,53;6,71]		-6,1 [-14,92;2,72]		-5,76 [-12,95;1,43]	
Part of a group	Yes	REF.		REF.		REF.		REF.	
	No	-2,86 [-11;5,28]		0,28 [-5,7;6,26]		-4,21 [-14,05;5,64]		-5,85 [-13,74;2,05]	
	Hospital-based	-17,59 [-27,92;-7,27]*		-7,62 [-15,21;-0,03]		-7,57 [-20,06;4,91]		-8,63 [-18,64;1,39]	
Dependency score (2016)		-0,04 [-0,09;0,01]		-0,01 [-0,05;0,02]		-0,02 [-0,08;0,03]		-0,03 [-0,07;0,02]	
Staff/ resident ratio (2016)		-7,55 [-18,26;3,15]		-1,07 [-8,65;6,5]		3,76 [-8,31;15,82]		0,46 [-9,42;10,33]	
RM officer	No	REF.		REF.		REF.		REF.	
	Yes	-3,19 [-11,38;5]		-1,13 [-6,86;4,59]		4,6 [-4,55;13,76]		1,45 [-6,08;8,99]	
Qualified RM expert	No	REF.		REF.		REF.		REF.	
	Yes	-12,77 [-20,11;-5,43]*		-6,43 [-11,79;-1,07]*		1,48 [-7,58;10,53]		1,63 [-5,76;9,03]	
External Quality and RM officer	No	REF.		REF.		REF.		REF.	
	Yes	-10,25 [-40,67;20,18]		-10,72 [-31,79;10,35]		-28,73 [-62,13;4,67]	**-31,08 [-61,21;-0,94]***	0,46 [-27,53;28,44]	
Established policy of ongoing improvement in Quality and RM	No	REF.		REF.		REF.		REF.	
	Yes	-2,27 [-10,49;5,94]		-4,99 [-10,57;0,59]	**-4,73 [-9,98;0,52]**	-8,78 [-17,72;0,16]		-3,21 [-10,71;4,29]	
Other strategic plans put in place by the NH	No	REF.		REF.		REF.		REF.	
	Yes	-4,31 [-12,27;3,66]		-5,09 [-10,53;0,35]		-10,26 [-18,87;-1,65]*		-1,99 [-9,35;5,37]	
Active Quality improvement approach	No	REF.		REF.		REF.		REF.	
	Yes	-11,42 [-24,13;1,3]		-6,62 [-15,54;2,31]		3,44 [-11,19;18,06]		-2,76 [-14,71;9,19]	
Active RM policy	No	REF.		REF.		REF.		REF.	
	Yes	-10,12 [-17,99;-2,24]*		-6,75 [-12,25;-1,25]*		-13,42 [-22,04;-4,8]*	**-10,49 [-18,73;-2,25]***	-6,56 [-13,98;0,85]	
% of variance explained (Adjusted R²)			**29,2%**		**14,7%**		**24,2%**		**NA**

Table A1 (suite)

		Dimension 5		Dimension 6		Dimension 7	
		Univariate	*Multivariate*	*Univariate*	*Multivariate*	*Univariate*	*Multivariate*
Geographic location	Loire-Atlantique	REF.		REF.		REF.	
	Vendée	6,53 [-0,3;13,36]		2,37 [-4;8,74]		-0,26 [-5,04;4,51]	
Beds	< 80	REF.		REF.		REF.	
	≥ 80	-5,15 [-12,05;1,75]	**-8,48 [-14,65;-2,32]***	-0,31 [-6,69;6,08]		-1,1 [-5,86;3,66]	
Legal status	Private ou public, independant or regional	REF.		REF.		REF.	
	Attached to a public hospital	-10,5 [-18,23;-2,76]*		-6,81 [-14,05;0,43]		-7,73 [-12,91;-2,54]*	
Specialised units	No	REF.		REF.		REF.	
	Yes	1,43 [-5,48;8,34]		0,79 [-5,69;7,26]		2,85 [-1,91;7,61]	
Part of a group	Yes	REF.		REF.		REF.	
	No	0,51 [-7,26;8,28]		1,71 [-5,29;8,71]		-1,15 [-6,13;3,83]	-1,95 [-7;3,11]
	Hospital-based	-6,3 [-16,15;3,56]		-5,98 [-14,86;2,91]		-9,22 [-15,54;-2,91]*	**-8,58 [-14,83;-2,33]***
Dependency score (2016)		0 [-0,04;0,05]		0,01 [-0,03;0,05]		0 [-0,03;0,03]	
Staff/ resident ratio (2016)		-7,71 [-17,1;1,69]		-1,45 [-10,17;7,27]		-1,22 [-7,73;5,29]	
RM officer	No	REF.		REF.		REF.	
	Yes	1,61 [-5,54;8,76]		2,74 [-3,19;8,67]		-2,74 [-7,64;2,16]	
Qualified RM expert	No	REF.		REF.		REF.	
	Yes	-9,45 [-16,02;-2,88]*	**-11,43 [-17,63;-5,24]****	-5,81 [-11,46;-0,15]*		-5,93 [-10,53;-1,34]*	
External Quality and RM officer	No	REF.		REF.		REF.	
	Yes	-18,72 [-44,82;7,39]	**-27,91 [-51,31;-4,51]***	0,85 [-21,29;23]		-1,36 [-19,71;16,99]	
Established policy of ongoing improvement in Quality and RM	No	REF.		REF.		REF.	
	Yes	-3,09 [-10,2;4,03]		-0,56 [-6,53;5,41]		-0,25 [-5,2;4,69]	
Other strategic plans put in place by the NH	No	REF.		REF.		REF.	
	Yes	-5,73 [-12,56;1,11]		-0,45 [-6,29;5,38]		0,06 [-4,77;4,9]	
Active Quality improvement approach	No	REF.		REF.		REF.	
	Yes	-5,23 [-16,51;6,05]		-10,85 [-19,88;-1,82]*	**-10,85 [-19,88;-1,82]***	-8,51 [-16,03;-0,99]*	**-7,48 [-14,92;-0,04]**
Active RM policy	No	REF.		REF.		REF.	
	Yes	-7,8 [-14,73;-0,87]*		-1,36 [-7,38;4,66]		-4,93 [-9,75;-0,11]	
% of variance explained (Adjusted R²)			**23,7%**		**7,5%**		**14,9%**

NA : No answer
RM : risk management
